# Minimum Vertex Cut with Reachable Set (MVCRS) Problem for Suppressing Botnet Propagation in IoT Networks: Complexity and Algorithms

**DOI:** 10.3390/s26082324

**Published:** 2026-04-09

**Authors:** Shingo Yamaguchi

**Affiliations:** Graduate School of Sciences and Technology for Innovation, Yamaguchi University, Ube 755-8611, Japan; shingo@yamaguchi-u.ac.jp

**Keywords:** IoT security, botnet containment, computational complexity, diffusion control, maximum flow minimum cut theorem, network resilience, reachable set optimization

## Abstract

This paper formulates the “Minimum Vertex Cut with Reachable Set” (MVCRS) problem as an optimization framework to suppress botnet propagation in networked systems, and clarifies its computational complexity and algorithmic solutions. Building a firewall to minimize damage is essential for addressing botnet propagation in Internet of Things (IoT) networks. We define the basic MVCRS problem as minimizing the sum of the weight of the deployed resources and the resulting propagation scope. While we demonstrate that the constrained version of the problem is NP-complete, we show that the fundamental trade-off optimization model can be solved in polynomial time by reducing it to the maximum flow–minimum cut problem. This provides a theoretical baseline for optimal resource allocation in cybersecurity. Experimental evaluations reveal the limitations of conventional heuristics. In community-structured networks, the degree-based greedy algorithm overlooks critical bridge nodes, yielding an optimality gap of up to 72.6% above the theoretical minimum cost. Conversely, our exact algorithm consistently guarantees the optimal minimum cost (a 0% gap) with high statistical stability across diverse topologies. Furthermore, it scales efficiently to solve 100,000-node IoT networks within practical time limits, proving to be a reliable and efficient foundation for botnet suppression in complex real-world systems.

## 1. Introduction

Controlling harmful diffusion phenomena is an urgent challenge in modern networked societies. Threats such as botnet proliferation, malware propagation, and cascading failures in communication networks share the common characteristic of propagating dynamically across complex graph structures [1,2].

The landscape of attacks and defenses in IoT and mobile networks has grown increasingly complex. On the attack side, adversaries employ advanced reconnaissance and side-channel techniques to identify vulnerabilities and achieve initial compromise. For instance, Li et al. [3] proposed FOAP, a fine-grained, open-world Android app fingerprinting method that identifies running applications from network traffic—a technique that could be exploited to target specific vulnerable apps. Furthermore, Ni et al. [4] demonstrated that physical-layer side channels, such as radio-frequency energy harvesting, can be abused to eavesdrop on mobile app activities. Once an initial node is compromised through such sophisticated vectors, the infection can rapidly propagate to form a massive botnet.

On the defense side, rapid patching is essential; for example, Sun et al. [5] introduced FLoRa, an energy-efficient and highly reliable beamforming-assisted firmware update scheme designed to secure resource-constrained LoRa networks. However, due to bandwidth bottlenecks and the massive scale of IoT networks, deploying immediate firmware updates to all devices simultaneously is often impractical. In scenarios where a zero-day exploit or initial compromise occurs before patches are fully deployed, dynamic network-level containment serves as a crucial layer of defense. By mathematically optimizing the isolation boundary, containment restricts the reachable damage scope and buys essential time until system-wide updates can be completed.

From a graph theory perspective, this problem has traditionally been addressed as the Minimum Vertex Cut Problem [6,7]. However, in real-world scenarios, the costs faced by the defense side extend beyond the simple number of cuts (number of resources invested). The total number of nodes still reachable from the initial source after applying cuts—the size of the “Reachable Set”—is the critical metric determining the ultimate scale of damage. While prior research has attempted to predict this outbreak scale using data-driven methods [8], a deterministic framework directly minimizing this scale from a structural perspective remains insufficiently established.

Existing research has widely adopted greedy approaches based on degree centrality or betweenness centrality due to their computational simplicity [9,10]. However, these methods rely on local topological information and do not guarantee an optimal trade-off considering the entire network. Meanwhile, the problem of minimizing damage under resource constraints (Influence Minimization) is generally considered computationally difficult (NP-hard) from a combinatorial optimization perspective, often leading to approximations based on submodularity [11,12].

This paper formulates the “Minimum Vertex Cut with Reachable Set (MVCRS)” problem, which minimizes the weighted sum of resource inputs and reachable set sizes, and clarifies its theoretical properties. The main contributions of this research are as follows.

1. Clarifying computational complexity: We prove that the constrained version of this problem (a decision problem) is NP-complete via polynomial-time reduction from the Clique problem.
2. Proposal of an Exact Algorithm: We devise a technique to reduce the basic model of the MVCRS problem to a maximum flow–minimum cut problem on a special auxiliary graph. This theoretically guarantees that an optimal solution can be derived in polynomial time.
3. Empirical Evaluation: Simulations on large-scale networks (up to 100,000 nodes) demonstrate that conventional greedy algorithms suffer an optimality gap of up to 72.6% in complex community structures. In contrast, the proposed method consistently guarantees the exact minimum cost (a 0% gap) with superior statistical stability and high scalability, computing optimal solutions within practical time limits.

The structure of this paper is as follows. Section 2 reviews related work, Section 3 rigorously defines the MVCRS problem. Section 4 and Section 5 present the computational complexity proof and details of the proposed algorithm, respectively. Section 6 presents the numerical simulation and discusses the limitations of conventional heuristics. Section 7 discusses the extension of the proposed model. Finally, Section 8 concludes the paper.

## 2. Related Work

This section reviews related work from five perspectives: (A) graph cut theory, (B) centrality metrics, (C) diffusion models, (D) critical node detection, and (E) IoT security, to clarify the position of our research.

A. Classical Graph Cut Theory and Vertex Cuts

The Minimum Vertex Cut Problem in graph theory seeks the smallest set of vertices to remove to separate a given source *s* and sink *t*. Based on Menger’s theorem [6], it can be solved in polynomial time using the maximum flow–minimum cut algorithm [7]. However, conventional minimum cut problems focus solely on minimizing “cut cost” and do not directly optimize the size of the remaining component (extent of damage) after the cut. The MVCRS problem proposed in this paper extends classical cut theory to real-world dynamic risk management [11,13] by simultaneously optimizing the trade-off between disconnection cost and the extent of remaining damage.

B. Greedy Algorithms Based on Centrality Measures

Strategies prioritizing the removal of nodes with high degree centrality or betweenness centrality in pandemic control and information diffusion suppression have been widely studied due to their computational simplicity [9,10]. Particularly in networks with community structures, removing edges or nodes that act as “bridges” between communities is considered effective [14,15]. However, these metrics are based on local topology or static graph structures, and there is no guarantee they can derive optimal solutions that dynamically consider the initial source location or the reachability of the spread [12,16].

C. Diffusion Models and Influence Minimization

As the inverse of the Influence Maximization problem [12] under stochastic diffusion models such as the Independent Cascade (IC) or Linear Threshold (LT) models, Influence Minimization aims to mitigate damage by isolating a specific set of nodes [17]. Traditionally, minimizing influence spread under resource constraints has been formulated as an NP-hard problem. Consequently, the literature has heavily relied on submodularity-based approximation algorithms [8,18,19], including advanced frameworks like CELF and IMM. While these methods achieve high approximation accuracy, their solutions are fundamentally derived from stochastic sampling and greedy strategies, which inherently fail to guarantee a globally exact optimal solution.

In stark contrast, the foundational Minimum Vertex Cut with Reachable Set (MVCRS) problem proposed in this paper introduces a fundamentally different paradigm. Instead of relying on approximations, our approach structurally embeds the evaluation metric—the size of the reachable set—directly into the capacities of a transformed flow network. This unique formulation allows us to leverage the Maximum Flow–Minimum Cut theorem to derive an exact, globally optimal solution in polynomial time, circumventing the conventional NP-hard limitations. Furthermore, in our evaluations, we contrast this with a degree-based greedy algorithm, a representative practical heuristic that focuses merely on local network topologies. This comparison highlights that while conventional heuristics often fall into local optima, our proposed exact method deterministically guarantees the true optimal containment strategy, offering a mathematically rigorous foundation for resilient IoT network management.

D. Critical Node Detection and Network Interdiction

Various containment strategies have also been explored in the fields of Critical Node Detection (CND) and Network Interdiction. However, the formulation of our MVCRS differs fundamentally from these approaches. CND typically does not assume a specific source of propagation and aims to minimize pairwise connectivity or the size of the maximum connected component across the entire network, making it generally NP-hard [20]. On the other hand, network interdiction employs a minimax game-theoretic approach, where the defender blocks nodes or edges to minimize the maximum traffic flow of the attacker between predefined specific nodes or to maximize the shortest path [21,22]. In contrast, the fundamental novelty of MVCRS lies in directly optimizing a dynamic “spread boundary” from a specific threat source without predefining the protected node (sink). That is, it simultaneously determines the optimal containment cut and the reachable set (damage range), shifting the focus from the general network fragmentation problem to a threat-source-specific dynamic damage mitigation problem.

E. Multi-layered Security Measures in IoT Networks

Security measures in IoT networks have evolved into multi-layered approaches, ranging from the protection of individual devices to coordinated defense across the entire network. For example, Nowroozi et al. proposed the RDFSA approach, which randomly selects features extracted by deep networks, thereby significantly reducing the transferability of adversarial attacks and improving model robustness [23]. Furthermore, regarding the efficiency of intrusion detection, Hadi et al. proposed GRAF-IDS, which applies graph theory-based clustering to federated learning, achieving high-precision threat detection while minimizing communication overhead [24].

While these studies focus on “individual node defense” and “high-efficiency detection,” the MVCRS model in this study optimizes “rapid containment at the topology level” immediately after a threat is detected through these methods. Specifically, it addresses the problem of how to limit the reachable set (scope of damage) at the lowest possible cost across the entire network—including nodes protected by RDFSA [23]—against attack sources identified by methods such as GRAF-IDS [24]. Integrating existing detection and defense technologies with the dynamic graph containment strategy proposed in this study is crucial for building a comprehensive defense framework against increasingly complex IoT threats.

## 3. Problem Formulation

This section formulates the proposed “Minimum Vertex Cut with Reachable Set (MVCRS) problem” and provides its mathematical definition for both the basic model and the constrained model.

### 3.1. Minimum Vertex Cut with Reachable Set (MVCRS) Problem

Phenomena such as botnet proliferation, malware propagation, and cascading failures in communication networks can be modeled as processes as processes originating from a specific vertex $v_{0}\in V$ on a graph G=(V,E) and propagating to adjacent structures over time. To minimize such damage, devising defense strategies that isolate appropriate nodes and contain propagation is essential. This paper defines the “Minimum Vertex-Cut with Reachable-Set (MVCRS) Problem,” which optimizes the tradeoff between the number of countermeasure resources deployed and the remaining damage extent, as follows.

**Definition 1**  (Minimum Vertex-Cut with Reachable-Set (MVCRS) Problem)**.** *Given an undirected graph G=(V,E), an initial infection source $v_{0}\in V$, and a weight coefficient w>0, the optimization problem of finding the vertex subset $C\subseteq V\setminus \{v_{0}\}$ that minimizes the cost function Cost(C) defined by the following equation is called the MVCRS problem.*

(1) $$\mathrm{Minimize}\;Cost\left(C\right)=w\cdot |C|+|R\left(v_{0},C\right)|$$

*Here, C is the set of vertices to be removed from G, and $R(v_{0},C)$ represents the set of vertices still reachable from $v_{0}$ in the induced subgraph G[V∖C] obtained by removing C from G (i.e., the diffusion range).*

While this definition assumes a single initial source $v_{0}$ to simplify the formulation and analysis, the model can be naturally extended to handle multiple sources, as will be discussed in Section 7.

**Figure 1:**
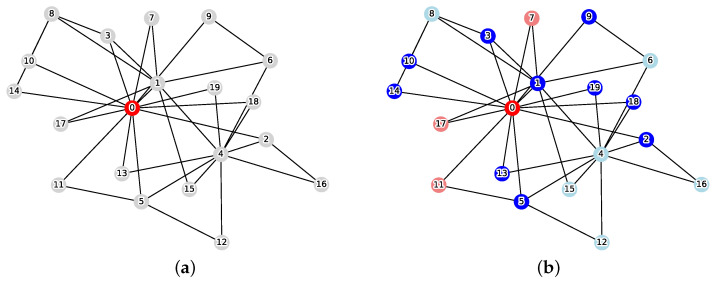
Instance of the MVCRS problem (|V|=20, $v_{0}=0$, w=1.0, red: infection source $v_{0}$, gray: normal, blue: vertex cut *C*, pink: reachable set *R*, cyan: protected vertices). (**a**) Initial state; (**b**) Optimal solution.

Figure 1 illustrates an example of this problem. When the initial infection source is $v_{0}=0$ and the weight coefficient is w=1.0, the solution that minimizes the cost function Cost(C) is C={1,2,3,5,9,10,13,14,18,19} and the reachable set is $R\left(v_{0},C\right)=\left\{0,7,11,17\right\}$. From Equation (1), the cost is calculated as follows:

Cost(C)=1.0×10+4=14.0

This problem exhibits the nature of a combinatorial optimization problem that simultaneously optimizes cut size and reachable set size.

### 3.2. Constrained MVCRS Problem

In real-world defense scenarios, practical constraints such as budget and personnel limitations often impose upper bounds on the resources that can be deployed at once (i.e., the number of nodes that can be removed). Considering such practical constraints, we define a model with an upper bound on the cut size |C| as the “Constrained-MVCRS Problem.”

**Definition 2**  (Constrained-MVCRS (Optimization) Problem)**.** *Given an undirected graph G=(V,E), an initial infection source $v_{0}\in V$, weight coefficients w>0, and an upper bound on vertex cut size $k\in Z^{+}$, find $C\subseteq V\setminus \{v_{0}\}$ that minimizes the cost function Cost(C) under the following constraints:*

$$\mathrm{Minimize}\;Cost\left(C\right)=w\cdot |C|+|R\left(v_{0},C\right)|$$

(2) subjectto|C|≤k

*If no C satisfying the condition exists, it is considered infeasible.*

**Figure 2:**
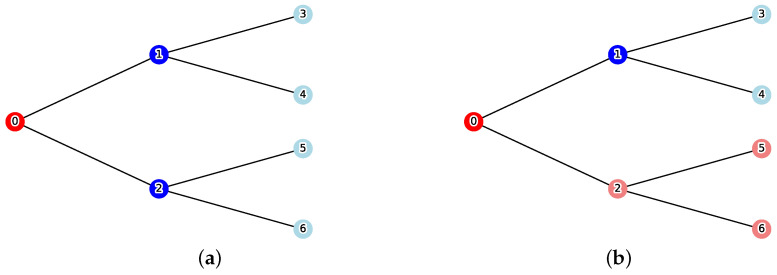
Comparison of optimal solutions between the unconstrained MVCRS and constrained MVCRS problems with $v_{0}=0$ and w=1.0. (**a**) Unconstrained MVCRS problem. The optimal solution is C={1,2}, yielding Cost=3.0. (**b**) Constrained MVCRS problem under the resource limit k=1. The optimal set is restricted to C={1}, resulting in an increased Cost=5.0.

Using Figure 2, let us examine the impact of the constraint. Under the conditions $v_{0}=0,w=1.0$, the optimal solution without the constraint is C={1,2}. From the reachable set $R\left(v_{0},C\right)=\left\{0\right\}$, the total cost is calculated as Cost(C)=1.0×2+1=3.0. However, under the resource upper bound constraint k=1, this solution becomes infeasible. The solution that satisfies the constraint while minimizing cost is C={1}. From the reachable set $R\left(v_{0},C\right)=\left\{0,2,5,6\right\}$, the total cost is calculated as Cost(C)=1.0×1+4=5.0.

Thus, in the constrained MVCRS problem, the optimal combination of nodes must be selected to minimize damage within limited resources. For networks with a large number of nodes, selecting this optimal combination is conjectured to be difficult to solve in polynomial time. The next section clarifies the computational complexity of the constrained MVCRS problem theoretically.

## 4. Computational Complexity

This section clarifies the computational complexity of the constrained MVCRS problem defined in the previous section. To discuss whether an algorithm exists that solves the optimization problem in polynomial time, we focus on the decision problem version (CMVCRS Decision Problem), which asks whether a feasible solution achieving a specific target cost exists.

We prove the complexity for the special case of the MVCRS decision problem where the weights of cut cost and damage cost are equal (w=1). In computational complexity theory, if a special case of a problem is NP-hard, then the general case allowing arbitrary parameters (w>0) is necessarily NP-hard as well. Therefore, proving NP-completeness under the constraint w=1 suffices to show that the general constrained MVCRS problem is NP-hard.

The proof proceeds in two steps:

- Step 1: Proof that the CMVCRS decision problem belongs to class NP (Lemma 1).
- Step 2: Proof that the CMVCRS decision problem is NP-hard (Theorem 1).

### 4.1. Preliminary

Before proceeding with the complexity argument, we define the target decision problem and the known NP-complete problem which reduces to the target.

**Definition 3** 
(CMVCRS Decision Problem with w=1)**.** *Given an undirected graph G=(V,E), an initial infection source $v_{0}\in V$, an upper bound on vertex cut size $k\in Z^{+}$, and an upper bound on target cost M>0. Determine whether there exists a vertex subset $C\subseteq V\setminus \{v_{0}\}$ satisfying both upper bounds simultaneously (Yes/No).*

**Definition 4**  (Clique Problem (CLIQUE) [25])**.** *Given an undirected graph $H=(V_{H},E_{H})$ and a positive integer k as input, determine whether a clique (a complete subgraph) of size k exists in H (Yes/No).*

### 4.2. Step 1: Proof of Belonging to Class NP

**Lemma 1.** 
*The CMVCRS decision problem (with w=1) belongs to class NP.*

**Proof.** 
We show that it is possible to verify in polynomial time whether a given candidate solution (a vertex set $C\subseteq V\setminus \{v_{0}\}$) is valid.

1. Cut size verification: Check that |C|≤k. This completes in O(|V|) time.
2. Verifying Reachable Set and Total Cost: Perform breadth-first search (BFS) starting from $v_{0}$ on the subgraph G[V∖C] to identify the reachable vertex set $R(v_{0},C)$. This search can be performed in O(|V|+|E|) time. Using the obtained |C| and $\left|R(v_{0},C)\right|$, compute the total cost $Cost\left(C\right)=|C|+|R\left(v_{0},C\right)|$ and verify that it is less than or equal to the target value *M*.

Since the above verification procedure completes in linear time O(|V|+|E|) relative to the graph size, this decision problem can be solved in polynomial time by a non-deterministic Turing machine and belongs to class NP. □

### 4.3. Step 2: Proof of NP-Hardness (Reduction from the Clique Problem)

We show that the Clique problem, a representative NP-complete problem, can be reduced to the CMVCRS decision problem in polynomial time.

**Figure 3:**
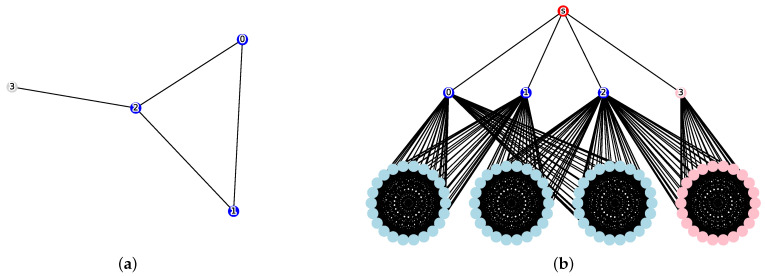
Illustration of the reduction process. (**a**) Original graph $H=(V_{H},E_{H})$ with a clique (blue) of size k=3. (**b**) Constructed graph *G*. For a target cost M=27, calculated as $M=k+1+\left(\right|V_{H}|-k)+\left(|E_{H}|-\frac{k(k-1)}{2}\right)\left(2k+\right|V_{H}{|}^{2})=27$, the vertex cut $C=\{v_{0},v_{1},v_{2}\}$ (blue) isolates the three large cliques (cyan) from the source *s* (red). This configuration verifies that Cost(C)=|C|+|R(s,C)|=3+24=27≤M.

**Construction 1.**  *Constructing an auxiliary graph G For any instance $H=(V_{H},E_{H})$ of the Clique problem and any positive integer k, construct an undirected graph G=(V,E) as an instance of the CMVCRS decision problem using the following procedure (a specific construction example is shown in Figure 3).*

*1.*  *Add a new vertex (initial infection source) $v_{0}$.*
*2.*  *Add each vertex $v\in V_{H}$ of the original graph H to G and create an edge connecting $v_{0}$ to each v.*
*3.*  *For each edge* $e\in E_{H}$ *of H, add a large clique* $K_{e}$*. Here, the number of vertices W in* $K_{e}$ *is set to a sufficiently large value to counteract the influence of the cut size upper bound k, as given by the following formula:* 
  (3) $$W=2k+|V_{H}{|}^{2}$$
*4.*  *For each edge* $e=\left(v,u\right)\in E_{H}$*, add an edge between its two endpoints* v,u *and all vertices belonging to the corresponding large clique* $K_{e}$.

*For the constructed graph G, the parameters for the CMVCRS decision problem are set as follows.*

- *Upper bound on vertex cut size: k.*
- *Target cost: M=k+B, where the baseline reachable set size is defined as follows.*
(4) $$B=1+\left(\right|V_{H}\left|-k\right)+\left(|E_{H}|-\frac{k(k-1)}{2}\right)W$$

The time required to construct this graph *G* is polynomial in the number of vertices and edges.

As illustrated in Figure 3, the construction is designed such that the existence of a *k*-clique in *H* corresponds directly to a “Yes” instance of the CMVCRS decision problem in *G*. Specifically, if a subset of vertices $C\subseteq V_{H}$ forms a clique of size *k* in the original graph *H*, selecting these vertices as the cut in *G* isolates all k(k−1)/2 large cliques $K_{e}$ associated with the edges of that clique. Since the weight *W* is defined to be sufficiently large (Equation (3)), the target cost *M* can be satisfied if and only if the number of isolated large cliques is maximized. This maximum is achieved precisely when the *k* chosen vertices are mutually adjacent in *H*, thereby rendering the corresponding $K_{e}$ components inaccessible from the source $v_{0}$. Consequently, the total cost Cost(C) meets the threshold *M*, validating that the reduction is both correct and polynomial in time.

**Lemma 2** 
(Equivalence)**.** *The existence of a clique of size k in graph H is equivalent to the existence of a vertex cut C in the constructed graph G satisfying the condition (|C|≤k and Cost(C)≤M).*

**Proof.**  (Proof of Necessity) Assume that there exists a clique $C_{H}\subseteq V_{H}$ of size *k* in the graph *H*. We select $C=C_{H}$ as the vertex cut in *G* (corresponding to the blue nodes in Figure 3b). First, since |C|=k≤k, the constraint on the cut size is satisfied. Next, we evaluate the size of the reachable set. Any vertex $v\in V_{H}\setminus C$ that is not included in the cut *C* remains reachable because it is directly connected to *s*. Since the selected vertex set *C* forms a clique of size *k* in the original graph *H*, there are exactly $\frac{k(k-1)}{2}$ edges within *C* in *H*. For each of these edges *e*, both endpoints are included in the cut *C*, and thus the corresponding large clique $K_{e}$ is completely disconnected from *s*. Conversely, for all other large cliques $K_{e}$, at least one endpoint remains in $V_{H}\setminus C$, making them reachable. Therefore, the size of the reachable set $\left|R(v_{0},C)\right|$ is the sum of *s* itself (1), the remaining vertices in $V_{H}$ ($|V_{H}|-k$), and the vertices in the reachable $K_{e}$ components ($\left(|E_{H}|-\frac{k(k-1)}{2}\right)W$), where *W* denotes the size of each large clique $K_{e}$, which exactly matches the baseline value *B*. Hence, Cost(C)=k+B=M, satisfying the target cost condition.

(Proof of Sufficiency) Assume that a vertex cut C⊆V∖{s} satisfies both |C|≤k and Cost(C)≤M=k+B. By our construction, the size *W* of each large clique $K_{e}$ is set to $2k+|V_{H}{|}^{2}$, which is strictly greater than the cut budget *k*. This implies that even if all *k* available cut units were spent within a single $K_{e}$, the component cannot be entirely disconnected; the only way to make a $K_{e}$ component unreachable from *s* is to include both of its corresponding endpoints $u,v\in V_{H}$ in the cut *C*. Let *m* denote the number of $K_{e}$ components that are made unreachable. We prove $m\geq \frac{k(k-1)}{2}$ by contradiction. Suppose $m\leq \frac{k(k-1)}{2}-1$. Under this assumption, the number of $K_{e}$ components that remain reachable is at least $|E_{H}|-\frac{k(k-1)}{2}+1$. The total size of the reachable set |R(s,C)| is then bounded from below by the sum of the source *s*, the remaining vertices in $V_{H}$, and these reachable $K_{e}$ components:

$$\left|R\left(s,C\right)\right|\geq 1+\left(\right|V_{H}|-|C|)+\left(|E_{H}|-\frac{k(k-1)}{2}+1\right)W$$

Recalling the baseline value $B=1+|V_{H}|-k+\left(|E_{H}|-\frac{k(k-1)}{2}\right)W$, the above inequality can be rewritten as

|R(s,C)|≥B+k−|C|+W

Substituting this into the cost constraint Cost(C)=|C|+|R(s,C)|≤k+B,

|C|+B+k−|C|+W≤k+B

By canceling *B* and *k* from both sides, and noting that the terms |C| and −|C| offset each other, we derive

W≤0

This clearly contradicts our construction where $W=2k+|V_{H}{|}^{2}>0$. Even using a more relaxed lower bound for the reachable set, the inequality W≤2k−|C| would still fail because W>2k. Therefore, we must have $m\geq \frac{k(k-1)}{2}$. On the other hand, a vertex set *C* of size |C| can cover at most $\frac{\left|C|(|C|-1\right)}{2}$ edges. Thus, the following chain of inequalities must hold:

$$\frac{k(k-1)}{2}\leq m\leq \frac{\left|C|(|C|-1\right)}{2}$$

Given the constraint |C|≤k, this is satisfied only when |C|=k and $m=\frac{k(k-1)}{2}$. This condition implies that the chosen *k* vertices cover exactly $\frac{k(k-1)}{2}$ edges, meaning the set *C* forms a clique of size *k* in the original graph *H*. □

By Lemmas 1 and 2, we have proven that the Constrained-MVCRS decision problem is NP-complete immediately.

**Theorem 1.** 
*The Constrained-MVCRS decision problem is NP-Complete.*

Since the decision problem version is NP-complete, we conclude that the Constrained-MVCRS optimization problem seeking its exact solution is NP-hard.

**Theorem 2.** 
*The Constrained-MVCRS optimization problem is NP-hard.*

Theorem 2 implies that the botnet containment problem remains computationally intractable when strict resource constraints are imposed. This gap between the constrained (NP-complete) and unconstrained (P) versions highlights that the feasibility of an exact solution depends on the formulation of the objective function. Consequently, the polynomial-time algorithm proposed in the next section is particularly valuable for scenarios where the primary goal is to find the most cost-effective balance between defense investment and potential damage, or for evaluating the performance of heuristics in constrained environments.

## 5. Polynomial-Time Algorithm for the Unconstrained MVCRS Problem

In the previous section, we proved that the constrained MVCRS optimization problem with a cut size upper bound *k* is NP-hard. In this section, we focus on the unconstrained MVCRS problem, which removes this constraint, and propose an algorithm demonstrating that an optimal solution for this problem can be derived in polynomial time. The core of our proposal lies in reducing the optimization problem on a weighted graph to the Minimum *s*–*t* Cut Problem in a directed network.

### 5.1. Reduction to the Minimum s–t Cut Problem

To solve the MVCRS Problem, we construct an auxiliary weighted directed network $G^{\prime }=\left(V^{\prime },E^{\prime }\right)$ from the original undirected graph G=(V,E).

**Construction 2**  (Building the Auxiliary Directed Graph $G^{\prime }$)**.** *Given an input graph G=(V,E), construct the directed graph $G^{\prime }$ using the following procedure.*

*1.*  *Vertex Splitting: For each vertex* v∈V *in G, create two vertices* $v_{in},v_{out}\in V^{\prime }$ *and add a directed edge* $\left(v_{in},v_{out}\right)$*. The capacity* $cap(v_{in},v_{out})$ *of this edge is set as follows:*
  $$cap\left(v_{in},v_{out}\right)=\left\{\begin{array}{cc} \infty & \mathrm{if}\;v=v_{0}\\ w & \mathrm{otherwise} \end{array}\right.$$
  *This setting mathematically corresponds to cutting an edge of capacity w with removing vertex v (*v∈C*) from the original graph.*
*2.*  *Definition of source vertex s and sink vertex t: The vertex ${v_{0}}_{in}$ corresponding to the initial infection source is defined as the source vertex s. Additionally, a new vertex is added to $V^{\prime }$ as the sink vertex t.*
*3.*  *Representation of Damage Cost (Reachability): For every vertex v∈V, draw a directed edge $\left(v_{out},t\right)$ from $v_{out}$ to the sink t, setting its capacity uniformly to* 1*. Cutting this edge means vertex v remains in the region on the s (i.e., $v_{0}$) side, and infection is reachable ($v\in R(v_{0},C)$).*
*4.*  *Maintaining Connectivity: For each undirected edge (u,v)∈E in the original graph, add directed edges $\left(u_{out},v_{in}\right)$ and $\left(v_{out},u_{in}\right)$ with capacity ∞ in $G^{\prime }$. This preserves the topology of G while setting the additional cost associated with moving between vertices to zero.*

**Figure 4:**
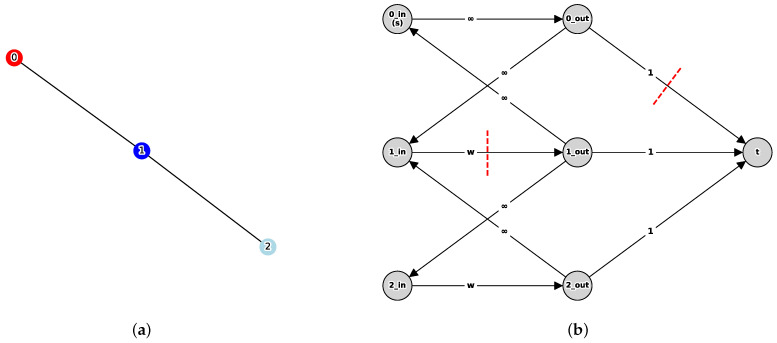
Transformation process from the original graph *G* to the auxiliary directed graph $G^{\prime }$. Each vertex *v* in graph *G* is split into $v_{in}$ and $v_{out}$, connected by a directed edge $\left(v_{in},v_{out}\right)$ (capacity *w*) (where $v=v_{0}$ implies capacity *∞*). The directed edge (capacity 1) from $v_{out}$ to the newly introduced sink vertex *t* represents the penalty cost for infecting that node (making it reachable from $v_{0}$). The connection (u,v) between vertices in the original graph is represented by two directed edges $\left(u_{out},v_{in}\right)$ and $\left(v_{out},u_{in}\right)$ (both with capacity *∞*). (**a**) Original graph *G*; (**b**) Auxiliary directed graph $G^{\prime }$.

Figure 4 illustrates the transformation process from the original undirected graph G=(V,E) to the auxiliary directed graph $G^{\prime }=\left(V^{\prime },E^{\prime }\right)$. Each vertex v∈V is represented in $G^{\prime }$ by a pair of nodes, $v_{in}$ and $v_{out}$, connected by a directed internal edge $\left(v_{in},v_{out}\right)$. The capacity of this edge is defined as *w*, corresponding to the cost of removing the vertex from the original graph. For the initial source vertex $v_{0}$, the capacity is set to *∞* to ensure it is not included in the minimum cut. To incorporate the reachability penalty, a sink vertex *t* is introduced, and for every vertex *v*, a directed edge $\left(v_{out},t\right)$ with capacity 1 is added. This unit capacity represents the cost incurred when *v* remains reachable from $v_{0}$. Furthermore, each original undirected edge (u,v)∈E is modeled by two directed edges, $\left(u_{out},v_{in}\right)$ and $\left(v_{out},u_{in}\right)$, both with infinite capacity. This construction ensures that the topological connectivity of *G* is preserved while preventing the selection of these edges in any finite s−t cut.

The fundamental methodological novelty of this study lies not merely in the choice of the objective function, but in the structural mapping of the reachable set optimization into a standard s−t minimum cut framework. Traditional minimum cut algorithms are strictly limited to separating a fixed, predefined source and sink. In dynamic threat propagation, however, the specific nodes that will ultimately be protected (the sink side) are unknown a priori, causing a combinatorial explosion. By introducing a super sink and connecting all potential target nodes to it with specific capacities, our approach mathematically redefines this combinatorial problem into a computationally efficient network flow problem. This structural mapping is what allows the algorithm to dynamically determine the optimal protection boundary that maximizes cost-effectiveness for the entire system in polynomial time, distinguishing our approach from conventional cut-based heuristics.

### 5.2. Theoretical Analysis

We prove that the minimum s−t cut in the directed graph $G^{\prime }$ constructed in the previous section is completely equivalent to the optimal solution of the MVCRS problem.

**Lemma 3** 
(Equivalence)**.** *The capacity of the minimum s−t cut in the auxiliary graph $G^{\prime }$ coincides with the minimum cost Cost(C) in the MVCRS problem.*

**Proof.** 
Let the vertex partition of $G^{\prime }$ by the minimum cut be (S,T) (where s∈S and t∈T). Define the vertex set $C\subseteq V\setminus \{v_{0}\}$ of the original graph based on this partition as follows:

$$C\overset{def}{=}\left\{u\in V|u_{in}\in S,u_{out}\in T\right\}$$

Since a minimum cut must have finite capacity, edges with capacity *∞* (the split edge of $v_{0}$ and connecting edges between vertices) are never included in the cut. Therefore, edges contributing to the cut capacity are limited to the following two types, and their respective contributions are uniquely determined as follows.

1. Vertex split edge $\left(u_{in},u_{out}\right)$ (capacity *w*): By definition, cutting this edge implies $u_{in}\in S$ and $u_{out}\in T$, which is equivalent to u∈C from the above definition. Thus, the total contribution of this edge class to the cut capacity is w·|C|.
2. Sink edge $\left(u_{out},t\right)$ (capacity 1): Cutting this edge implies, by definition, that $u_{out}\in S$ (since t∈T). The constraint that edges with capacity *∞* are not cut ensures reachability within *S* is preserved. Therefore, $u_{out}\in S$ is equivalent to the original graph having an undeleted vertex u∉C reachable from the initial source $v_{0}$ ($u\in R(v_{0},C)$). Thus, the total contribution of this edge class to the cut capacity is $1\cdot |R(v_{0},C)|$.

The sum of these two types yields the total capacity of the minimum cut as $w\cdot |C|+|R(v_{0},C)|$. This coincides exactly with the objective function Cost(C) of the MVCRS problem. Thus, finding a minimum cut in $G^{\prime }$ is equivalent to minimizing the cost of the MVCRS problem. □

**Theorem 3** 
(Polynomial-Time Solvability)**.** *The MVCRS problem can be solved in polynomial time to find an optimal solution.*

**Proof.**  By Lemma 3, the MVCRS problem reduces to the minimum s−t cut problem on the directed auxiliary graph $G^{\prime }$. From the construction procedure, the number of vertices in graph $G^{\prime }$ is $|V^{\prime }|=2|V|+1$, and the number of edges is at most $|E^{\prime }\left|=|V|+2|E|+|V\right|$. These are both of linear order O(|V|+|E|) relative to the input size. By the Max-flow Min-cut theorem, the minimum cut problem is equivalent to the maximum flow problem. Using efficient algorithms such as Dinic’s algorithm [26], the maximum flow problem can be solved in time complexity $O(|V^{\prime }{|}^{2}|E^{\prime }|)$. Therefore, the MVCRS problem can be solved in polynomial time relative to the input graph size. □

This algorithm offers a novel approach to large-scale optimisation by aggregating all nodes in the network into a super sink. In contrast, conventional s−t minimum cut algorithms were limited to separating fixed pairs of points. By performing this mapping, we have transformed the problem of “wide-area optimisation of reachable sets” into one that can be solved using the efficient computational framework of the maximum flow algorithm. This approach’s greatest significance lies in its ability to rapidly and mathematically identify a protective boundary (cut) that maximises the system’s overall cost-effectiveness in scenarios involving dynamic threat propagation where the specific targets to be protected are not predefined. This formulation introduces a new paradigm in diffusion control theory that reconciles computational complexity with practical requirements.

## 6. Numerical Simulation

### 6.1. Setup

This section presents the evaluation results of numerical simulations conducted to verify the effectiveness of our polynomial-time exact algorithm for the MVCRS problem. The objectives of this evaluation are as follows: (1) to verify the cost minimization capability compared to existing heuristic methods; (2) to evaluate the scalability of computation time for large-scale networks.

A. Dataset

Real-world IoT network datasets with large-scale and detailed topological structures, which are the subject of this study, are extremely limited in their public availability due to corporate security and privacy concerns. Furthermore, many existing datasets, such as BoT-IoT [27], provide only traffic data. Therefore, in this experiment, we adopted a hybrid model that reflects the complexity of real-world IoT environments, in addition to two basic logical models designed to comprehensively evaluate the algorithm’s characteristics.

1. Barabási–Albert (BA) Model: A graph exhibiting scale-free characteristics, as seen in the Internet. This model evaluates the influence of high-degree nodes (hubs) on the propagation of malicious traffic and the effectiveness of defenses against them.
2. Stochastic Block (SB) Model: A graph with a community structure featuring dense internal connections. This model verifies the risk of explosive infection spread within clusters and the containment effect achieved by blocking bridge nodes connecting communities.
3. Hierarchical Hybrid Topology: A model integrating a wide-area mesh backbone with local access networks to more accurately reflect the complexity of real-world IoT networks. The Watts–Strogatz model [28] was applied to the backbone network, generating random shortcuts with a 30% probability in the ring-shaped connections between hubs to reproduce small-world characteristics. Furthermore, the local access networks connected to the hubs consist of a 50-50 mix of star-shaped networks (modeling home Wi-Fi, etc.) and complete graph networks (modeling direct communication such as Zigbee or Bluetooth mesh).

B. Comparison Method

To evaluate the performance of the proposed method, we adopted two heuristic methods based on representative centrality metrics as baselines. Both methods sort all nodes (excluding the infection source $v_{0}$) according to a specific criterion, add them to the vertex cut *C* in descending order, and calculate the MVCRS cost (w|C|+|R|). They then output *C* at the point where the minimum cost is achieved as the solution. Note that both methods use ascending order of the number of hops from the source node $v_{0}$ as the first sorting criterion.

- Greedy-Degree: A method that uses descending order of degree as the second criterion. This strategy prioritizes isolating high-degree nodes, and since it requires only sorting by node degree, it is extremely fast. It demonstrates high defensive performance in scale-free networks dominated by a few large hubs. On the other hand, it has the weakness of easily overlooking key “bridges” that connect communities—even if they have low degree—and is prone to allowing the spread of infection to other areas.
- Greedy-Betweenness: A method that uses descending betweenness centrality as the second criterion. This strategy prioritizes blocking bottlenecks where traffic is concentrated and is effective in networks with cluster structures. However, since it requires calculating the shortest paths for all node pairs, the computational cost is very high, and scalability issues remain for application to large-scale networks. Furthermore, it has the drawback of easily falling into redundant double blocking, as adjacent equivalent bridges remain high on the list immediately after a specific bridge is blocked.

C. Experimental Environment

The experiments were conducted on a system running Ubuntu 22.04, an Intel Xeon @ 2.20 GHz, and 12 GB of RAM (Python 3.12). For the maximum flow search in the proposed method, we used a standard implementation of Dinic’s algorithm [26].

### 6.2. Evaluation of Cost Minimization Capability

We present experimental results on the cost minimization capabilities of three network models (BA, SB, and hierarchical hybrid). We measured the defense cost (MVCRS cost) calculated by each method as an evaluation metric and compared the trends when the node isolation weight parameter *w* (w∈{1,3,5,7,9,11}) was varied. The number of trials was 100.

Since the proposed method is a deterministic algorithm that always derives the theoretical exact solution, its cost serves as the baseline for evaluating other heuristic methods. To quantify how much a heuristic method deviates from the theoretical minimum cost, we use the “Optimality Gap” defined as follows:

(5) $$\mathrm{Optimality}\;\mathrm{Gap}\;\left(\%\right)=\frac{{\mathrm{Cost}}_{\mathrm{heuristic}}-{\mathrm{Cost}}_{\mathrm{proposed}}}{{\mathrm{Cost}}_{\mathrm{proposed}}}\times 100$$

A gap of 0% indicates that the heuristic method successfully reached the optimal solution, while a larger gap represents the degree of unnecessary resource consumption or propagation damage.

A. Evaluation on the BA Model

**Figure 5:**
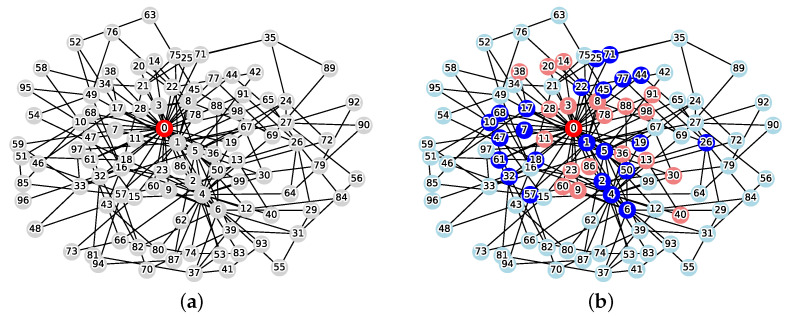
Application example of the proposed method on the BA model (|V|=100, average degree d=4, weight w=2, red: infection source $v_{0}$, gray: normal, blue: vertex cut *C*, pink: reachable set *R*, cyan: protected vertices). (**a**) Initial state; (**b**) Application result.

Figure 5 shows an example of applying the proposed method to a scale-free BA model (with |V|=100 nodes and an average degree of d=4) where the weight coefficient for defense costs is set to w=2.

**Figure 6:**
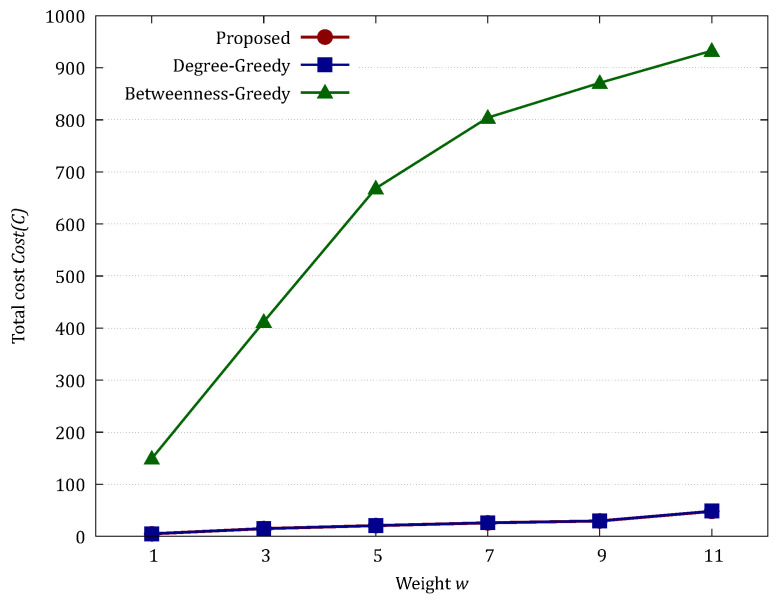
Comparison of total cost in the BA model.

Table 1 and Figure 6 show the evaluation results for larger nets (|V|=1000, d=4, w=2).

In the BA model, which is characterized by a scale-free structure, both the Proposed method and the Degree-Greedy method exhibited nearly identical performance. As shown in Table 1, the Optimality Gap of the Degree-Greedy method remained extremely low, ranging from 0.00% to 1.52% across all values of *w*. This indicates that in topologies dominated by a few massive hubs, the local node degree serves as a highly effective proxy for identifying the global min-cut. In such specific environments, the heuristic approach can fortuitously achieve near-optimal results. However, the Proposed method consistently derived the theoretical minimum cost with lower standard deviation (e.g., SD = 55.67 vs. 58.95 at w=11), ensuring the most stable and reliable defense regardless of the specific network instance. In contrast, the Betweenness-Greedy method showed a massive Optimality Gap (exceeding 1800%), demonstrating that betweenness centrality is unsuitable for this cost-minimization problem due to its tendency to select redundant nodes.

B. Evaluation on the SB Model

**Figure 7:**
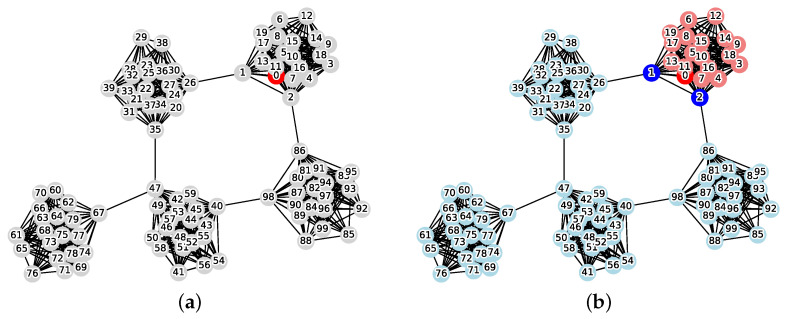
Application example of the proposed method on the SB model (|V|=100, 5 groups, weight w=2, red: infection source $v_{0}$, gray: normal, blue: vetex cut *C*, pink: reachable set *R*, cyan: protected vertices). (**a**) Initial state; (**b**) Application result.

Figure 7 illustrates the application of the proposed method to an SB model with a community structure (|V|=100, w=2, five groups), using $p_{in}=0.8$ and $p_{out}=0.0005$ as the intra- and inter-community edge probabilities, respectively.

**Figure 8:**
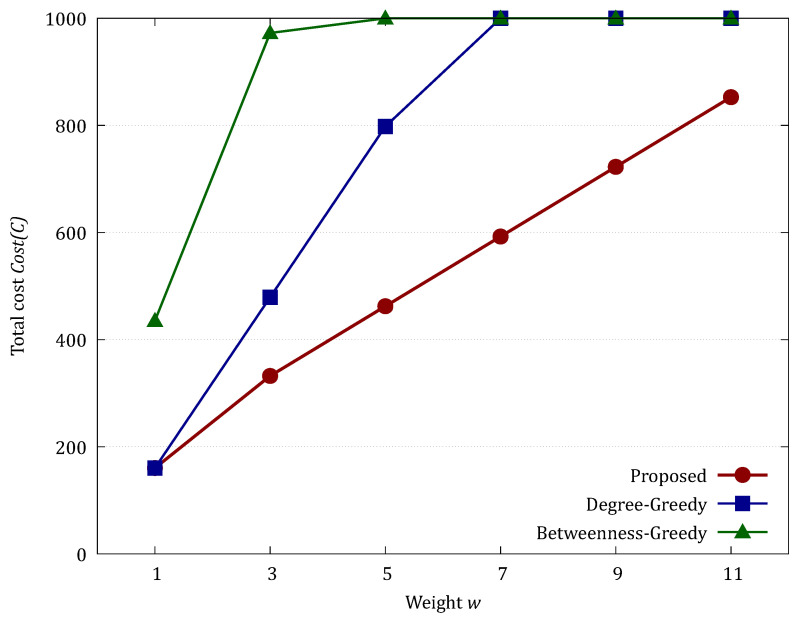
Comparison of total cost in the SB model.

Table 2 and Figure 8 show the evaluation results for larger nets (|V|=1000, five groups, $p_{in}=0.8$ and $p_{out}=0.0005$, weight w=2).

The results for the SB model highlight the true advantage of the Proposed method in networks with community structures. While the Degree-Greedy method was optimal at w=1, its performance deteriorated sharply as *w* increased, with the Optimality Gap reaching 44.22% at w=3 and peaking at 72.58% at w=5. This significant gap occurs because the greedy approach prioritizes high-degree nodes within clusters but fails to identify the inconspicuous “bridge nodes” that connect different communities. Consequently, the heuristic method allows the infection to leak into other clusters, eventually hitting the cost upper bound (1000) at w≥7. The Proposed method, by contrast, utilizes the max-flow min-cut principle to capture these global bottlenecks, maintaining significantly lower costs (Mean = 852.78 at w=11) and providing a robust defense that remains effective even when isolation costs are high.

C. Evaluation on the Hierarchical Hybrid Topology

**Figure 9:**
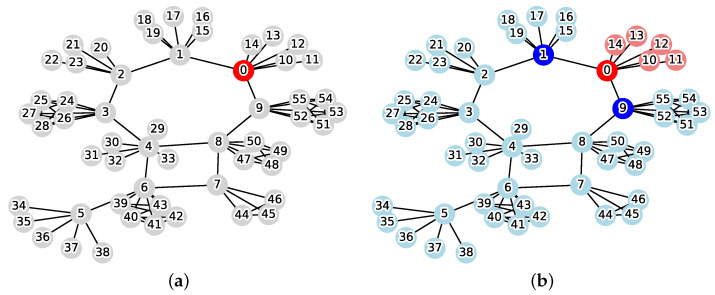
Application example of the proposed method on the Hierarchical Hybrid Topology (|V|=56, average number of end devices per hub = 5, weight w=2, red: infection source $v_{0}$, gray: normal, blue: vertex cut *C*, pink: reachable set *R*, cyan: protected vertices). (**a**) Initial state; (**b**) Application result.

Figure 9 illustrates the application of the proposed method to an example of applying the proposed method to the hierarchical hybrid topology with a weight coefficient of w=2 (|V|=56, average number of end devices per hub = 5).

**Figure 10:**
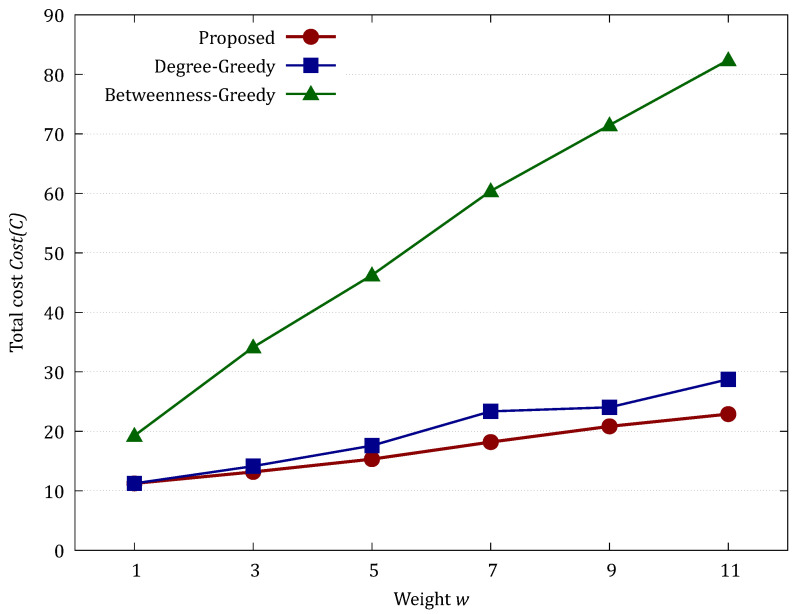
Comparison of total cost in the Hierarchical Hybrid Topology.

Table 3 and Figure 10 show the evaluation results for larger nets (|V|=1000, average number of end devices per hub = 50, w=2).

In the hierarchical hybrid topology, which more closely mimics complex real-world IoT environments, the Proposed method demonstrated superior stability and reliability. Compared to the Proposed method, the Betweenness-Greedy method showed a significantly large Optimality Gap, exceeding 250% at w=11, suggesting that betweenness centrality fails to effectively capture the optimal cut in such heterogeneous structures. A key observation is the difference in the standard deviation of the results. As *w* increased, the Degree-Greedy method exhibited extreme fluctuations; at w=11, it produced a maximum cost of 254 and a large standard deviation (SD = 39.92), indicating its effectiveness is highly dependent on the random topology of the local network. In contrast, the Proposed method consistently identified the optimal vertex cut, maintaining a much tighter distribution (Max = 59, SD = 11.58 at w=11). The Optimality Gap of the Degree-Greedy method reached up to 28.35% at w=7, confirming that even in hybrid networks, local heuristics cannot guarantee the level of protection provided by our exact algorithm. This stability is crucial for practical deployments requiring consistent defense performance.

### 6.3. Computational Time Scalability

To evaluate the practicality of the method for large-scale networks, we varied the number of vertices |V| from 100 to 100,000 in the aforementioned BA model and measured the computation time. The number of trials was 10. We excluded the Greedy (Betweeness) method from the comparison because its computational time increased rapidly compared to the other methods.

**Figure 11:**
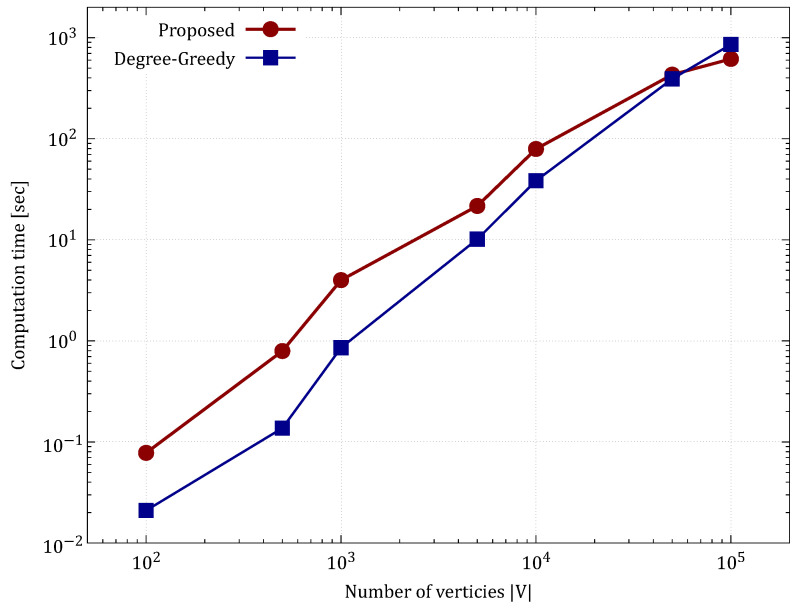
Evolution of computation time (sec) with the number of vertices in the BA model.

The results are shown in Table 4 and Figure 11.

Table 4 and Figure 11 show the evaluation results. The computation time of the proposed method showed behavior that was generally close to linear with respect to the increase in the number of vertices. While the theoretical worst-case time complexity of this algorithm is $O(|V{|}^{2}|E|)$, it was demonstrated that the maximum flow search using Dinic’s algorithm converges extremely rapidly on sparse graphs resembling real-world network topologies.

Notably, on a large-scale topology with |V| = 100,000, the proposed method, which derives an exact solution (616.49 s), outperformed the computational speed of the heuristic greedy method (857.49 s). This indicates that while the greedy method’s neighborhood search cost increases significantly as the number of nodes grows, the proposed method’s flow network model functions extremely efficiently. This result strongly suggests that the proposed method is applicable for constructing responsive and optimal defense postures even against real-world networks ranging from tens of thousands to hundreds of thousands of nodes.

## 7. Discussion

Although the basic MVCRS model assumes a single infection source and uniform costs, its framework is highly extensible without increasing computational complexity.

First, the single-source assumption can be easily relaxed by introducing a “super-source” in the auxiliary graph. By connecting the super-source to all known infected nodes with infinite-capacity edges, the multi-source problem is reduced to the basic model, maintaining polynomial-time solvability.

Second, the model can accommodate heterogeneous node importance and defense costs. By assigning node-specific weights to the edges in the auxiliary graph (e.g., setting the capacity of edges toward *t* as a node-specific weight instead of 1), the algorithm can prioritize the protection of critical infrastructure.

Finally, while the current static model provides a rigorous foundation, extending it to dynamic or stochastic environments—where infection probabilities are considered—represents an important future direction. This study bridges the gap between theoretical diffusion control and practical IoT security by providing a flexible optimization platform.

## 8. Conclusions

This paper proposed the Minimum Vertex Cut with Reachable Set (MVCRS) problem to optimize the trade-off between defense and damage costs against botnet propagation in IoT networks. Our theoretical analysis proved the constrained version is NP-hard using a polynomial-time reduction from the Clique problem. Conversely, we developed an exact polynomial-time algorithm for the fundamental unconstrained problem by reducing it to a minimum s−t cut on a vertex-split auxiliary digraph. Experimental evaluations across diverse topologies reveal the inherent limitations of conventional heuristics; in community-structured networks, degree-based greedy algorithms overlook critical “bridge nodes”, resulting in an optimality gap of up to 72%. In contrast, our proposed method consistently guarantees the exact minimum cost (a 0% gap) with superior statistical stability. Furthermore, our approach scales efficiently to 100,000-node networks using Dinic’s algorithm, providing a mathematically robust and highly scalable foundation for suppressing botnet propagation in complex IoT environments.

The objective function $Cost\left(C\right)=w|C|+|R\left(v_{0},C\right)|$ formulated in this study provides critical strategic implications for optimal resource allocation in practical IoT security. The weight coefficient *w* naturally models the relative cost—such as system downtime, operational impact, or computational overhead—of deploying countermeasures like isolating compromised devices or blocking network ports. By tuning this weight, network administrators can mathematically derive an optimal containment strategy that minimizes the propagation of IoT botnets while avoiding excessive network disconnection, thereby maximizing the protected operational area.

Future challenges include adapting to dynamic networks with constantly changing topologies [29] and extending the optimization model to handle multiple simultaneous initial infection sources [30]. Furthermore, with practical network operations in mind, developing distributed algorithms that operate autonomously using only local node information, without requiring global topology information, is also a critical research theme [31].

## Figures and Tables

**Table 1:** Comparison of total cost and Optimality Gap in the BA model.

Method	*w*	Mean	Gap (%)	Max	Min	Median	SD
Degree- Greedy	1	4.76	0.00	22	3	3	3.47
	3	14.83	0.54	178	7	10	21.22
	5	20.60	0.39	176	11	16	21.41
	7	26.06	0.89	323	15	15	34.83
	9	29.55	0.27	109	19	19	19.16
	11	48.65	1.52	485	23	34	58.96
Betweenness- Greedy	1	149.38	3038.2	258	5	157	55.56
	3	412.11	2693.9	689	19	445	135.78
	5	668.50	3157.8	1000	16	722.5	218.19
	7	804.24	3013.6	1000	29	929	274.65
	9	871.06	2855.7	1000	55	1000	234.62
	11	932.80	1846.5	1000	89	1000	166.48
Proposed	1	4.76	-	22	3	3	3.47
	3	14.75	-	174	7	10	20.79
	5	20.52	-	172	11	16	21.10
	7	25.83	-	300	15	15	32.87
	9	29.47	-	109	19	19	19.03
	11	47.92	-	451	23	34	55.67

**Table 2:** Comparison of total cost and Optimality Gap in the SB model.

Method	*w*	Mean	Gap (%)	Max	Min	Median	SD
Degree- Greedy	1	160.42	0.00	171	149	161	5.27
	3	479.26	44.22	511	445	481	15.81
	5	798.10	72.58	851	741	801	26.34
	7	1000.00	68.76	1000	1000	1000	0.00
	9	1000.00	38.37	1000	1000	1000	0.00
	11	1000.00	17.26	1000	1000	1000	0.00
Betweenness- Greedy	1	435.29	171.34	495	321	440	31.62
	3	972.62	192.67	1000	675	1000	57.02
	5	1000.00	116.24	1000	1000	1000	0.00
	7	1000.00	68.76	1000	1000	1000	0.00
	9	1000.00	38.37	1000	1000	1000	0.00
	11	1000.00	17.26	1000	1000	1000	0.00
Proposed	1	160.42	-	171	149	161	5.27
	3	332.32	-	362	303	335	12.59
	5	462.44	-	522	405	466	25.00
	7	592.56	-	682	507	598	37.44
	9	722.68	-	842	609	730	49.89
	11	852.78	-	1000	711	862	62.30

**Table 3:** Comparison of total cost and Optimality Gap in the Hierarchical Hybrid Topology.

Method	*w*	Mean	Gap (%)	Max	Min	Median	SD
Degree- Greedy	1	11.25	0.00	26	2	8.5	9.38
	3	14.15	7.36	79	4	11.5	12.42
	5	17.60	14.81	121	6	14.5	17.28
	7	23.36	28.35	155	8	19	27.86
	9	24.04	15.35	235	10	20	28.26
	11	28.75	25.55	254	12	20	39.92
Betweenness- Greedy	1	19.27	71.29	41	2	20	9.89
	3	34.14	159.03	65	4	35	14.29
	5	46.26	201.76	88	6	46	17.69
	7	60.40	231.87	111	8	59.5	25.36
	9	71.47	242.95	139	10	74.5	32.20
	11	82.41	259.87	163	23	78	33.29
Proposed	1	11.25	-	26	2	8.5	9.38
	3	13.18	-	33	4	11.5	9.60
	5	15.33	-	45	6	14.5	9.92
	7	18.20	-	44	8	19	9.89
	9	20.84	-	55	10	20	11.25
	11	22.90	-	59	12	20	11.58

**Table 4:** Evolution of computation time (sec) with the number of vertices in the BA model.

Method	|V|	Mean	Max	Min	Median	SD
Degree- Greedy	100	0.02	0.04	0.01	0.02	0.00
	500	0.14	0.21	0.07	0.14	0.00
	1000	0.86	2.24	0.30	0.71	0.65
	5000	10.15	22.73	5.02	9.51	5.23
	10,000	38.43	50.35	16.85	43.72	12.85
	50,000	391.19	872.49	169.06	374.68	208.11
	100,000	857.49	1208.28	403.22	990.15	302.77
Proposed	100	0.08	0.10	0.04	0.09	0.00
	500	0.79	1.68	0.17	0.71	0.45
	1000	4.00	10.14	0.80	4.30	2.98
	5000	21.64	77.30	3.61	15.47	21.66
	10,000	79.24	121.21	18.10	93.95	40.67
	50,000	430.97	1505.89	86.85	329.56	418.71
	100,000	616.49	1189.62	135.64	707.50	358.06

## Data Availability

The original contributions presented in this study are included in the article. Further inquiries can be directed to the corresponding author.

## Abbreviations

The following abbreviations are used in this manuscript:

IoT	Internet of Things
BA	Barabási–Albert
SB	Stochastic Block
